# Cost-Effectiveness of Pre-exposure Prophylaxis Among Men Who Have Sex With Men in China: A Systematic Review

**DOI:** 10.3389/fpubh.2022.809268

**Published:** 2022-06-21

**Authors:** Yuanqi Mi, Yuhong Zeng, Peicheng Wang, Mengge Zhou, Feng Cheng

**Affiliations:** ^1^School of Nursing, Peking Union Medical College and Chinese Academy of Medical Sciences, Beijing, China; ^2^Department of Epidemiology, College of Preventive Medicine, Army Medical University (Third Military Medical University), Chongqing, China; ^3^Vanke School of Public Health, Tsinghua University, Beijing, China; ^4^School of Medicine, Tsinghua University, Beijing, China

**Keywords:** human immunodeficiency virus (HIV), China, men who have sex with men (MSM), pre-exposure prophylaxis (PrEP), homosexual, cost-effectiveness (CE)

## Abstract

**Objective:**

Men who have sex with men in China meet the definition of the population at “substantial risk” of contracting human immunodeficiency virus (HIV) according to the World Health Organization; therefore, initiating pre-exposure prophylaxis (PrEP) is recommended for this population. Lack of convincing evidence on cost-effectiveness has resulted in the lack of large-scale PrEP implementation at a national level. The objective of this review is to assess the cost-effectiveness of pre-exposure prophylaxis implementation among men who have sex with men in China.

**Methods:**

The following databases were used to search studies of pre-exposure prophylaxis in China in both English and Chinese: PubMed, Embase, the China National Knowledge Infrastructure (CNKI), and the Wanfang Database. Inclusion criteria included pre-exposure intervention, year for publication (2007–2021), setting (China), and cost-effectiveness estimation.

**Results:**

Seven studies were identified. We found that pre-exposure prophylaxis is only cost-effective among men who have sex with men without prioritization with at least a 5.5% reduction in the market price in the models. Pre-exposure prophylaxis is potentially cost-effective when using the latest market price, combined with other preventive programs or delivered to the population with a high risk of HIV exposure.

**Conclusion:**

Our study identifies key considerations in cost-effectiveness evaluation: cost assumptions, implementation coverage, and targeted population. The scarce evidence available is not comparable to some extent. However, combined with the latest market and policy reform, the cost-effectiveness of PrEP could be achieved as estimated by the underlying model of the included studies. Consequently, it calls for more standard and transparent modeling studies that include the latest drug types and market prices.

## Introduction

The estimated number of people living with human immunodeficiency virus (HIV) in China reached 1.25 million by 2018 (1). National data in 2015 indicated that 30% of new HIV infections in China were transmitted by men who have sex with men (MSM). However, the rate in Beijing was as high as 75%, which showed a regional inconsistency (2). Global estimation showed that MSM had almost 20 times greater odds of acquiring HIV compared to the general population (3), mainly because of potential risk factors such as multiple sex partners and unprotected anal intercourse (4, 5).

Pre-exposure prophylaxis (PrEP) is an important biomedical tool for preventing HIV transmission. The clinical safety of PrEP was first demonstrated by Peterson et al. (6) in 2007. Later, studies around the world (7), including a large-scale cohort study in western China (8), demonstrated the efficacy of both daily PrEP and on-demand PrEP among MSM. Currently, more than 50 countries and regions have approved the use of PrEP. However, the cost-effectiveness (CE) of using PrEP remains debated. Several studies have assessed the CE of PrEP and suggested that PrEP is more cost-effective in populations at a substantially high risk of HIV exposure (9) and in low-income countries (10). The targeted population of PrEP varies depending on guidelines from different countries and regions, but all of them mainly considered the following several aspects: no HIV infection, recent (last 6 or 12 months) sexually transmitted infection (STI), recent use of post-exposure prophylaxis (PEP), defined high-risk population (the number of their sex partners, sex with HIV+ partners, commercial sex, and inconsistent condom use) (11–13). For example, Taiwan has a risk index to quantitatively assess HIV risk levels among MSM (14).

The World Health Organization (WHO) recommended that, in the absence of PrEP, high-risk people with an HIV incidence of >3 per 100 person-years should be considered for PrEP (15). However, an HIV incidence >2 per 100 person-years was considered sufficient to warrant offering oral PrEP in the recommendations issued by the International Antiviral Society at an expert panel in 2014 (16). According to a survey, China reached an HIV incidence of 5.6/100 among MSM by 2016 (17). However, large-scale PrEP implementation at a national level is currently unavailable. However, related research and policies have been promoting PrEP. In 2018, the China Medical University initiated a real-world study in four cities to evaluate the efficacy of the two different PrEP strategies (3). The Chinese Center for Disease Control and Prevention initiated a PrEP preventive pilot work involving 54 MSM in seven provinces using Truvada from 2018 to 2019 (18). In 2019, the Implementation Plan for Controlling HIV Transmission (2019–2022) (19) recommended preventive pilot work expansion for MSM and PrEP-related policy establishment. In August 2020, Truvada became the first PrEP drug approved by the National Medical Products Administration (17). Meanwhile, the Expert Consensus on pre-exposure prophylaxis for HIV in China provided medication guidance (17). By 2021, real-world studies have been conducted in multiple cities of China, including Shenyang, Beijing, Shenzhen, Chongqing, Xinjiang, Sichuan, Guangxi, Hong Kong, and Taiwan. Under a positive policy environment, PrEP is becoming accepted by an increasing number of MSM. Studies have indicated a relatively low but increasing awareness of PrEP, ranging from 3 to 43.1% among MSM in China (20–23), as well as a strong willingness to use PrEP, especially oral PrEP, which ranges from 65.8 to 84.9% (24, 25). In addition, a clinical trial of PrEP in western China suggested that MSM have a relatively high adherence rate of 64.29% (23).

With more evidence suggesting that PrEP could be a promising approach to HIV prevention among MSM in China, new concerns have been raised. For example, with the implementation of PrEP, users might reduce condom use and increase the number of sex partners, thus increasing the risk of acquiring other STIs (20). Additionally, financial burdens seem to be a problem, as only 6.8% of MSM in Chengdu were willing to pay the current market price ($3,396 per person/year) (22). Furthermore, expert consensus on pre-exposure prophylaxis for HIV in China recommended the targeted population using international guidelines (17). However, research suggested that directly utilizing current international guidelines to screen out MSM who self-identified as interested in PrEP could lead to misallocation of resources, as the distribution of MSM and their willingness for PrEP usage in different regions of China are uneven (11).

With limited resources, policymakers need more strategic and systematic information to make decisions. To our knowledge, there has been no systematic review identifying the CE of PrEP implementation among MSM in China. In this study, we systematically reviewed the evidence on the CE of different PrEP implementations to achieve the most economical outcome. We aim to identify the optimal regimen along three aspects: the most cost-effective PrEP coverage, the definition of high-risk MSM for the Chinese population, and the most cost-effective type of PrEP.

## Materials and Methods

We performed a systematic review of the published literature adhering to the PRISMA 2020 guidelines for reporting systematic reviews (26).

### Information Sources

We conducted a systematic search of electronic databases from February 2007 (since PrEP was proven to be safe in 2007) to May 2021, including PubMed, Embase, the China National Knowledge Infrastructure (CNKI), and the Wanfang Database. We restricted the language to Chinese and English.

### Search Strategy

The search strategies are presented in Table 1 and were assessed independently by two investigators (YQM and MGZ).

**Table 1:** Search strategies.

**Database**	**Access date**	**Searching category**	**Search strategy**
PubMed	22 May 2021	All journals	(“MSM”[Title/Abstract] OR “men who have sex with men”[Title/Abstract] OR “gay”[Title/Abstract] OR “homosexuality, male”[MeSH Terms]) AND (“preexposure prophylaxis”[Title/Abstract] OR “PrEP”[Title/Abstract]) AND (“Cost”[Title/Abstract] OR “economic*”[Title/Abstract]) AND “China”[All Fields]
Embase	17 August 2021	All journals	(msm OR gay OR 'men who have sex with men'/exp OR homosexual) AND (prep OR 'preexposure prophylaxis'/exp) AND ('cost'/exp OR 'cost' OR 'cost allocation' OR 'cost sharing' OR 'costs and cost analysis' OR 'deductibles and coinsurance' OR economic) AND 'china'/exp
CNKI	22 May 2021	Medicine & Public Health (Journal, Featured journal, Doctoral dissertation, Master dissertation)	((TKA= ‘男同’) or (TKA=‘男男性行为’) or (TKA=‘MSM’)) and ((TKA=‘暴露前预防’) or (TKA=‘PrEP’)) and ((TKA=‘经济’) or (TKA=‘成本’)) ((TKA=‘nantong’) or (TKA=‘nannanxingxingwei’) or (TKA=‘MSM’)) and ((TKA=‘baoluqianyufang’) or (TKA=‘PrEP’)) and ((TKA=‘jingji’) or (TKA=‘chengben’))
Wanfang Database	22 May 2021	All journals (Journal articles, Dissertations)	题名或关键词:(男同) or 摘要:(男同) or 题名或关键词:(MSM) or 摘要:(MSM) or 题名或关键词:(男男性行为) or 摘要:(男男性行为者)) and (题名或关键词:(暴露前预防) or 摘要:(暴露前预防) or 题名或关键词:(PrEP) or 摘要:(PrEP)) and (题名或关键词:(成本) or 关键词:(成本) or 题名或关键词:(经济) or 关键词:(经济)) (subject or key words:(nantong) OR abstract:(nantong) or subject or key words:(MSM) OR abstract:(MSM) OR subject or key words:(nannanxingxingwei) OR abstract:(nannanxingxingweizhe)) and (subject or key words:(baoluqianyufang) OR abstract:(baoluqianyufang) OR subject or key words:(PrEP) OR abstract:(PrEP)) and (subject or key words:(chengben) OR key words:(chengben) OR subject or key words:(jingji) OR key words:(jingji))

“*/exp ”- search strategy: Searches your term (or maps to the preferred Emtree term) and related narrower or children terms*.

### Inclusion/Exclusion Criteria

Full-text journal articles were included if: (1) The study included MSM or focused on services aimed at MSM; (2) The interventions involving PrEP were compared to conventional care or other interventions; (3) The study was conducted in China; (4) The study was published in 2007 or after; (5) The study included MSM who were HIV-negative and were able to use PrEP-related drugs; (6) Analytic models were applied to evaluate the health economic outcomes of PrEP implementation; and (7) The study included an economic evaluation of cost–benefit, cost-utility, or CE analysis. No restrictions were made on the type of models, assumptions of the models, mode of transmission, or the impact (effectiveness) metric chosen.

The exclusion criteria included the following: (1) quality-adjusted life years (QALYs) or disability-adjusted life years (DALYs) were not reported, or (2) the articles were reviews, protocols, letters, editorials, conference abstracts, poster presentations with insufficient details, or case reports.

### Data Extraction and Analysis

We systematically reviewed the literature on CE analysis that compared PrEP to a comparison case (no intervention or other intervention). We reported CE studies that used cost per HIV infection averted (HIA), cost per life-year (LY) saved, or cost per DALY/QALY averted/gained as the main outcome variable. The threshold of three times gross domestic product (GDP) per capita by the WHO-CHOICE project was used as a benchmark to determine the CE of each implementation (27). These standards depended on the GDP per capita, indicating that the general public will pay more than one times the GDP per capita or up to three times the GDP per capita. To summarize the information obtained from the individual studies, we created Tables 2–4 to systematically organize study characteristics that were independently abstracted from relevant studies, such as data concerning outcomes, details of the interventions, assumptions of costs, and impacts. Due to the heterogeneity of each study's design or model assumptions, we were unable to categorize studies by intervention or conduct a meta-analysis. However, to achieve more comparability, we converted the economic outcomes of each study into US dollars according to the exchange rate in each year.

**Table 2:** Study characteristics.

**Study reference**	**Type of analytical model**	**CHEERS (%) (quality classification)**	**Type of PrEP**	**Population targeted**	**Location**	**Demographic characteristics**	**HIV incidence/prevalence among MSM**	**Time-frame**	**Intervention**	**Coverage**	**Additional information**
Zhong et al. (27)	Markov model	22 (good quality)	Drug kind: Tenofovir (TDF); Type of dosing: oral; Frequency: event-driven.	MSM aged more than 14 years old	China	MSM current number: 10,000,000. Frequency of insertional sex: 1 time/week.	n/a	30 y	Promote HIV PrEP among MSM in China by means of TDF entering pharmacies (purchasing drugs with doctors' prescriptions) for marketing.	Percent of the population group using PrEP: 82.46%.	Per capita disposable income (2017): 25974 RMB ($4018.5). (2017 US$)
Wei et al. (28)	Dynamic compartmental model	20 (good quality)	Drug kind: TDF/FTC (Truvada); Type of dosing: oral; Frequency: daily.	MSM aged 14–64 years old	China	MSM: casual partners: 6/y (number of casual sexual behaviors: 14.4/y,); steady partners-MSP: 2/y (number of steady sexual behaviors: 51.2/y). HRMSM: the number of casual partnerships which MSM with MCP has is 1.5 times that of regular people: 9/y; the number of steady partnerships which MSM with MSP has is 1.5 times that of regular people: 3/y)	Prevalence: 6.3% in 2011.	10 y, 2016–2025	PrEP implementation among MSM.	Percent of the population group using PrEP: 10%, 20%, 30%, 40%, 50%, 60%, 70%, 80%, 90%.	Sensitivity analysis included the influence of three factors: effectiveness of PrEP (20%, 70%), cost of TDF (50% and 90% reduction) and behavioral change (20% reduction on condom using and 20% increase of sex partners). Per capita GDP(2016):¥54,000($8126).(2016 US$)
Fan et al. (29)	Markov model	21 (good quality)	Drug kind: TDF (Tenofovir); Type of dosing: oral; Frequency: daily.	MSM	China	n/a	Prevalence: 5.3% in 2016.	20 y	Intervention 1: standard HIV intervention strategies (including HIV testing, risk-reducing counseling, condom distribution, STI management). Intervention 2: daily oral PrEP (only TDF).	Not specified.	Per capita GDP(2016): ¥53,980($8126).(2016 US$) Sensitivity analysis conducted.
Zhang et al. (30)	Deterministic compartmental model	24 (excellent quality)	Intervention 1:Drug kind: TDF/FTC (Truvada); Type of dosing: oral; Frequency: daily.	High-risk MSM by definition	China	8.2 million Chinese men were estimated as sexually-active MSM (2% of sexually-active male population) and 2.5 million high-risk MSM were PrEP-eligible. “High-risk MSM” are defined as those who satisfied at least one of the following: (1) reported more than 10 anal sex partners in the past 6 months; (2) reported condomless anal sex in the past 6 months; (3) diagnosed with an STI in the past 6 months. 30% (20–40%) of Chinese MSM as “high- risk.”	Prevalence: 8% in 2016.	20 y	Intervention 1: daily Truvada.	Percent of the population group using PrEP: 20, 50, and 80%.	Per-capita GDP: $8126 (2016) Sensitivity analysis: adjusted the proportion of HRMSM from 20 to 40% (due to the large number of scenarios, only data from scenarios mentioned in the “results” of this article is presented in the outcome)
			Intervention 2:Drug kind: TDF/FTC (Truvada); Type of dosing: oral; Frequency: event-driven.						Intervention 2: on-demand Truvada.		
			Intervention 3:Drug kind: Tenofovir (TDF); Type of dosing: oral; Frequency: daily.						Intervention 3: daily TDF.		
			Intervention 4: Drug kind: TDF/3FC; Type of dosing: oral;Frequency: daily.						Intervention 4: daily TDF+3TC.		
Wong et al. (31)	Deterministic compartmental model	21 (good quality)	Drug kind: not specified; Type of dosing: oral;Frequency: daily.	All MSM aged 15–64. We assumed the presence of assortative mixing pattern, i.e., high-risk susceptible MSM mixed with high-risk infected MSM. 57% of MSM were deemed to be low-risk.	Hong Kong, China	A MSM is classified as belonging to the low-risk category if he has lower partner exchange rate ( ≤ 8 sexual partners per year), or high-risk if there has been higher partner exchange rate (>8 sexual partners per year). Low-risk MSM were assumed to be in serial monogamy partnership while high-risk MSM were in random mixing partnership, corresponding with the low and high frequency of partner exchange in the model.	n/a	5 y, 2017–2021	Intervention A: different coverage of PrEP involving both low-risk and high-risk MSM (i.e., non-targeting approach) or involving high-risk MSM only (i.e., targeting approach); and treatment initiation (minimum 90% from 2017, when test and treat was implemented).	Percent of the population group using PrEP: 10, 30, and 90%.	Sensitivity analysis conducted.
									Intervention B: a high rate of diagnosis.		
Li et al. (31)	Deterministic compartmental model	24 (excellent quality)	Drug kind: TDF/FTC (Truvada); Type of dosing: oral; Frequency: daily.	MSM aged 15–64 years old	China	The total population of MSM was divided between high-risk and low-risk at a ratio of 1:4 (20% of the population was high-risk) in a total population of 3,625,000. This division was based on annual sexual partnerships, with high-risk men having 15 per year and low-risk men having 2.6, for a balanced average of 5 partnerships per year.	Prevalence: 3.4% among MSM aged 15–64 in 2005.	20 y, 2017–2037	Intervention 1: test-and-treat strategy.	Percent of the population group using PrEP: 25, 50, and 75%.	Interventions that cost less than per capita gross domestic product (i.e., 15,943 Int.$) per QALY gained are defined as very cost-effective. Sensitivity analysis conducted.
									Intervention 2: PrEP targeting HRMSM. Intervention 3: expanded annual VCT.		
Hu et al. (32)	The risk-equation model	23 (good quality)	Drug kind: TDF/FTC (Truvada); Type of dosing: oral; Frequency: daily.	The partner of MSM aged 18 years or older and had a diagnosis within 6 months of HIV infection.	Shenyang, China	216 MSM with EHI were identified in the cohort study.	Incidence: 5.61/100 person-years; prevalence: 10%.	36 months	Intervention 1: ART for HIV+ MSM;	Percent of the population group using PrEP: 100%.	Sensitivity analyses were used to evaluate the impact of critical parameters on cost-effectiveness: HIV prevalence, HIV incidence, PrEP effectiveness, PrEP drug cost per day, ART drug cost per day, and life expectancy after ART initiation.
									Intervention 2: PrEP for their HIV- partners.		

*n/a, not applicable*.

*Not included: not experimented in the scenario*.

*Not specified: not clarified in the article*.

*CHEERS: Consolidated Health Economic Evaluation Reporting Standards is a 24-item checklist with a maximum score of 24. Studies that fulfilled 24 of the items were classified as excellent quality, those that fulfilled between >18 and <24 of the items were classified as good quality, those that fulfilled between >12 and ≤ 18 were classified as moderate quality, and those that fulfilled ≤ 12 were classified as low quality (ART, antiretroviral therapy; PrEP, pre-exposure prophylaxis; QALY, quality-adjusted life years; HIV, human immunodeficiency virus; MSM, men who have sex with men; GDP, gross domestic product; VCT, volunteer counseling and testing; STI, sexually transmitted infection)*.

Extracted information on the study design included the type of model utilized, study quality classification, type of PrEP, timeframe, setting and population, background HIV prevalence or incidence, demographic characteristics, a detailed description of interventions compared in the studies, and its coverage in Table 2. We also tabulated data on the effectiveness of PrEP, adherence or behavioral change expected after introduction of PrEP, and economic assumptions including expected drug cost, other service costs, indirect medical costs, antiretroviral treatment (ART) costs averted, and discount rates in Table 3. The prioritized group for which PrEP was specifically offered, CE results, and the conclusion of each scenario are presented in Table 4.

**Table 3:** Cost and impact assumptions.

**Study reference**	**Drug costs**	**Service costs**	**Indirect medical cost**	**ART costs**	**Discount rate**	**Effectiveness**	**Adherence**	**Behavior change (while on PrEP)**
Zhong et al. (27)	The cost of TDF is ¥85/bottle/30 tablets (purchased from CDC, not the market price in China, 52 weeks a year), the frequency of sexual behavior is once a week, and the cost is ¥143 ($20.5) per person-y (2017 US$).	Not included.	Transport: the number of drug purchases is 3.2 times per y, the one-way transportation cost is ¥8, and the cost is ¥256 ($36.6) per person-y (2017 US$). Working hours lost staff fee: ¥78.7 ($11.3) per person-y. (2017 US$)	¥36,795 ($5,263.9) per person-y (2017 US$).	3% annual discount rate	PrEP was assumed to be 100% effective when the adherence reached 80% or above.	Optimistic, 94%; Neutral, 80%.	Not included.
Wei et al. (28)	¥55,380 ($8,327.8) per person-y (2016 US$) (sensitivity analysis: 50% reduction or 90% reduction on the cost of TDF/FTC).	Non-HIV related health care cost, HIV testing cost, cost of follow-ups, cost of behavioral psychological counseling, liver and kidney function testing cost: ¥5,904 ($887.8) per person-y.	Not included.	¥31,200($4,691.7) per person-y.	Undiscounted	PrEP was assumed to be 44% effective (sensitivity analysis: 20, 70%)	Yearly PrEP drop-out rate: 1.3%.	Not included (sensitivity analysis: 20% increase of sex partners, 20% reduction on condom using).
Fan et al. (29)	Intervention 2(annual cost of daily oral TDF): ¥12,000 ($1,804.5) per person-y (2016 US$)	Intervention 1(standard HIV intervention strategies): ¥474 ($71.28) per person-y.	Not included.	HIV/AIDS related treatment cost: ¥22,000 ($3,308.3) per person-y.	5% annual discount rate	Not specified.	Not specified.	Not included.
Zhang et al. (30)	PrEP annual cost (daily Truvada) (2016 US$): $3457.1 per person-y. PrEP annual cost (On-demand Truvada) (2016 US$): $1843.8 per person-y. PrEP annual cost (daily TDF) (2016 US$): $785.7 per person-y. PrEP annual cost (Daily TDF+3TC) (2016 US$): $1,039.5 per person-y.	HIV, and STI screening: $95 per person-y.	Not included.	1st-line treatment: $473 per person-y. 2st-line treatment: $1,488 per person-y.	3% annual discount rate	PrEP was assumed to be 80% effective.	Mean duration of PrEP use of 20 years (sensitivity analysis: adjusted the duration of PrEP use before usage fatigue between 2 and 10 years).	Not included.
Wong et al. (33)	PrEP annual cost (high adherence in 87.5% usage, daily oral HKD188 (~$24) per dose) (2016 US$): $7,703 per person-y. PrEP annual cost (low adherence in 38% usage, daily oral HKD188 (~$24) per dose) (2016 US$): $3,345 per person-y. Plan A-market price for PrEP drug (annual cost of $7,800 at the end of 2017). Plan B-generic price for PrEP drug (annual cost of $519 per person-y). Plan C-zero cost for PrEP drug.	Testing cost for PrEP (HIV per visit, and creatinine, syphilis, CT/NG once per year): $3345 per person-y.	Not included.	ART annual cost for HIV- infected: $16,761 per person-y; cost for CD4 and viral load measurement (4 times per year): $410 per person-y.	3.5% annual discount rate	Effectiveness of PrEP was 70% in high adherence with>75% usage, and 23% in low adherence PrEP.	High adherence was defined as 87.5% usage and low adherence as 38% usage of daily oral PrEP. As it was obvious that low adherence PrEP would not be cost-effective, only scenarios with high adherence PrEP had been developed in the cost-effectiveness analysis.	Drop-out rate of PrEP usage: 20% for both high and low adherence. Annual rate of changing PrEP adherence: 20% from high to low adherence; 10% from low to high adherence. Duration of stable sexual partnership: 57% of low risk group.
Li et al. (31)	Drug Costs (2017 US$): US$6,909 per person-y (including PrEP drug cost and clinics cost).	Non-HIV related health care cost: $764 per person-y, cost of HIV ELISA antibody test: $25 per person-y, cost of confirmatory western blot test: $85 per person-y, cost of behavior counseling: $28 per person-y.	Not included.	Annual cost of ART: $6,540.	3% annual discount rate	PrEP was assumed to be 60% effective (sensitivity analysis: Optimistic, 90%; Neutral, 60%; Pessimistic, 30%)	100%.	Not included (sensitivity analysis: assuming all PrEP users in scenario 2–10 completely stopped using condoms with their sex partners).
Hu et al. (32)	Drug Costs (2017 US$): US$3,706 per person-y.	HIV screening; STIs testing and treatment; regular medical care: $347 per person-y.	Transport, working hours lost staff fee: $237 per person-y.	Estimated cost of ART within 12 months post-infection: $3,612 (3,233–3,991) per person-y. Estimated cost of ART within 36 months post-infection: $7,019 (6,308–7,730) per person-y.	Undiscounted	PrEP was assumed to be 90% effective.	100%. (all sexual partners were assumed to take daily PrEP until their partners reached undetectable VL)	Not included.

*2016 US$: 6.65. [Jan.]*

*2017 US$: 6.99. [Jan.]*

*Data source: China Foreign Exchange Trade Center, http://www.chinamoney.com.cn/chinese/forsddshis/index.html?dataType=6 (ART, antiretroviral therapy; PrEP, pre-exposure prophylaxis; HIV, human immunodeficiency virus; CDC, centers for disease control; MSM, men who have sex with men; VL, viral load; STIs: sexually transmitted infection; CT/NG, chlamydia detection/ Neisseria gonorrhoeae detection; ELISA, enzyme-linked immune sorbent assay; GDP, gross domestic product; CD4, cluster of differentiation 4; person-y: person-year)*.

**Table 4:** Cost-effectiveness estimation by scenario.

**Study reference**	**Base comparison scenario**	**Scenario Description: prioritization**	**Cost/QALY gained (ICER)**	**Cost/LY saved**	**Cost/DALYs averted**	**Cost/HIV infection averted**	**Conclusion**
Zhong et al. (27) (2017 US$)	No PrEP. Current HIV prevention strategies were included.	Scenario 1: optimistic: 94% adherence, no prioritization.	S1: -¥19,000/QALY gained. (-$2,718.2)	S1: ¥152,500. ($21,816.9)	not included	S1: ¥6144.6. ($879.1)	With higher adherence (no <80% is cost-effective) among MSM, PrEP implementation leads to higher cost-effectiveness.
		Scenario 2: neutral: 80% adherence, no prioritization.	S2: -¥14,700/QALY gained. (-$2,103)	S2: ¥117,900. ($16,867)		S2: ¥7798.0. ($1,115.6)	
Wei et al. (28) (2016 US$)	No PrEP. Current HIV prevention strategies were included.	S1–9: 10%-90% PrEP coverage, 44% PrEP effectiveness, no prioritization.	S1–S9: ¥513,242-¥855,299 ($77,179.2-$128,616.4). [S2–20% coverage: ¥293,717($44,168.0)]	Not included	Not included	Not included	Targeting HRMSM with MCP or 50% HRMSM with MSP is cost-effective. Marginal revenue will decrease if the coverage of PrEP increases. Only if the drug cost decrease to 60% of the current market price or the effectiveness of PrEP increase to 70% when PrEP is cost-effective among MSM without prioritization. PrEP is cost-effective among HRMSM with MCP when PrEP has a 70% effectiveness or higher. PrEP is cost-effective among HRMSM with MSP when PrEP has a 25% effectiveness or is less than 80% of current market price.
		S10–18: 10%-90% PrEP coverage, 44% PrEP effectiveness, HRMSM with MCP targeted.	S10–S18: ¥214,319-¥348,198 ($32,228-$52,360.6) [s11–20% coverage: ¥100,940 ($15,178.9)]				
		S18–27: 10%-90% PrEP coverage, 44% PrEP effectiveness, HRMSM with MSP targeted.	S19–S27: ¥97,404-¥158,649 ($14,647.2-$23,857.0) [S20–20% coverage: ¥152,808 ($22,978.6)]				
		S28: 20% PrEP coverage, 20% PrEP effectiveness, no prioritization.	S28: ¥810,035 ($121,809.8)				
		S29: 20% PrEP coverage, 70% PrEP effectiveness, no prioritization.	S29: ¥400,346 ($60,202.4)				
		S30: 20% PrEP coverage, 20% PrEP effectiveness, HRMSM with MCP targeted.	S30: ¥360,097 ($54,149.9)				
		S31: 20% PrEP coverage, 70% PrEP effectiveness, HRMSM with MCP targeted.	S31: ¥152,680 ($22,959.4)				
		S32: 20% PrEP coverage, 20% PrEP effectiveness, HRMSM with MSP targeted.	S32: ¥173,853. ($26,143.3)				
		S33: 20% PrEP coverage, 70% PrEP effectiveness, HRMSM with MSP targeted.	S33: ¥59,707 ($8,978.5)				
		S34: 20% PrEP coverage, 44% PrEP effectiveness, no prioritization with 20% increase of sex partners.	S34: ¥651,486. ($97,967.8)				
		S35: 20% PrEP coverage, 44% PrEP effectiveness, no prioritization with 20% reduction of condom using.	S35: ¥593,566 ($89,258.0)				
		S36: 20% PrEP coverage, 44% PrEP effectiveness, no prioritization with 50% reduction on the cost of TDF.	S36: ¥229,951 ($34,579.1)				
		S37: 20% PrEP coverage, 44% PrEP effectiveness, no prioritization with 90% reduction on the cost of TDF.	S37: ¥0. ($0)				
Fan et al. (29) (2016 US$)	No Prep. Current HIV prevention strategies were not included.	Scenario 1: standard HIV intervention strategies (including HIV testing, risk-reducing counseling, condom distribution, STI management), no prioritization,	S1: ¥12,597.3 ($1,894.3)	Not included	Not included	Not included	Only the standard intervention strategy is cost-effective. The combination strategies (scenario 2) is not cost-effective unless TDF has a 5.5% reduction on current price.
		Scenario 2: standard HIV intervention strategies (including HIV testing, risk-reducing counseling, condom distribution, STI management), daily oral PrEP (only TDF), no prioritization.	S2: ¥123,626.0 ($18,590.4) (ICER=¥162,395.24 ($24,420.3) compared to scenario 1)				
Zhang et al. (30)	No intervention among HRMSM.	30%% HRMSM, mean duration of PrEP use of 20 years S1–S3: daily Truvada, 2018–2037, 20%/50%/80%.	Not included	Not included	S2: $49,400;	S2: $113,300;	At 50% coverage, both daily TDF and daily TDF/3TC is cost-effective no matter when the PrEP implementation started. The cost of PrEP needs to be below a threshold of $1,700 per person-y to be cost-effective. The cost of Truvada would have to be cut by about 50% under scenario using daily Truvada (Changing the parameters in the sensitivity analysis do not change the findings for cost-effectiveness of various PrEP implementation strategies modeled.)
		S4–S6: daily Truvada, 2023–2037, 20%/50%/80%.			S5: $67,400;	S5: $140,800;	
		S7–S9: Intermittent Truvada, 2018–2037, 20%/50%/80%.			S8: $26,400;	S8: $60,600;	
		S10–S12: Intermittent Truvada, 2023–2037, 20%/50%/80%.			S11: $36,100;	S11: $75,400;	
		S13–S15: daily generic TDF, 2018–2037, 20%/50%/80%.			S14: $11,400;	S14: $26,100;	
		S16–S18: daily generic TDF, 2023–2037, 20%/50%/80%.			S17: $15,500;	S17: $32,500;	
		S19–S21: daily generic TDF/3TC, 2018–2037, 20%/50%/80%.			S20: $15,000;	S20: $34,400;	
		S22–S24: daily generic TDF/3TC, 2023–2037, 20%/50%/80%.			S23: $20,400.	S23: $42,800.	
		(Sensitivity analysis: Condition 1: 20% HRMSM, mean duration of PrEP use of 5 years; Condition 2: 40% HRMSM, mean duration of PrEP use of 5 years; Condition 3: 30% HRMSM, mean duration of PrEP use of 2 years; Condition 4: 30% HRMSM, mean duration of PrEP use of 10 years.)					
Wong et al. (33)	No PrEP. HIV prevalence would increase from 0.08 in 2011 to 0.19 in 2021, while HIV incidence (per 100 person-years) would increase from 1.1 to 1.6. The number of locally acquired new infections would increase from 395 in 2011 to 604 in 2021.	S1–S3: non-targeting A, 10%, 30%, 90%, plan A.	S1–S3: $1,745,524-$2,115,619.	Not included	Not included	Not included	PrEP would not be cost-effective with the current market drug price for PrEP, whereas test-and-treat without PrEP was the most cost-effective intervention. Scenario 30 has the minimum ICER among PrEP strategies using the current market price. If assuming plan B or C, strategies targeting 30% HRMSM for PrEP has the minimum ICER. In the case of Hong Kong, a 93% reduction of the drug cost (Plan B, annual USD519/person in 2017) is desirable in order to demonstrate PrEP's cost-effectiveness at 30% coverage.
							(Sensitivity analysis showed that the increase in the number of HRMSM on high-adherence PrEP, inclusion of low-risk MSM and expansion of PrEP coverage would avert more infections.)
		S4–S6: non-targeting A, 10%, 30%, 90%, plan B.	S4–S6: $243,483-$298,518.				
		S7–S9: non-targeting A, 10%, 30%, 90%, plan C.	S7–S9: $137,545-$170,358.				
		S10–S12: targeting A, 10%, 30%, 90%, plan A.	S10–S12: $1,583,136-$2,162,072.				
		S13–S15: targeting A, 10%, 30%, 90%, plan B.	S13–S15: $219,862-$306,779.				
		S16–S18: targeting A, 10%, 30%, 90%, plan C.	S16–S18: $123,710-$175,926.				
		S20: plan B.	S20: $396,874.				
		S21–S23: B+non-targeting A, 10%, 30%, 90%, plan A.	S21–S23: $929,215-$1,985,645.				
		S24–S26: B+non-targeting A, 10%, 30%, 90%, plan B.	S24–S26: $268,915-$305,830.				
		S27-S29: B+non-targeting A, 10%, 30%, 90%, plan C.	S27–S29: $180,901-$261,863.				
		S30–S32: B+targeting A, 10%, 30%, 90%, plan A.	S30–S32: $668,940-$1,366,821.				
		S33–S35: B+targeting A, 10%, 30%, 90%, plan B.	S33–S35: $247,356-$331,116.				
		S36–S38: B+targeting A, 10%, 30%, 90%, plan C.	S36–S38: $168,400-$307,290.				
Li et al. (31)	We projected a base-case model that assumed current Chinese HIV treatment guidelines were followed for the next 20 years with no change in testing uptake and treatment entry rates.	Scenario 1: Test-and-treat strategy fully compliant with the WHO 90-90-90 recommendations (annual testing rates of 90% for all MSM, with an ART utilization rate of 90% for all diagnosed PLWH, and 90% ART effectiveness).	s1: $1,754.	Not included	Not included	Not included	Test and treat strategy in scenario 1 is the most cost-effective approach. When resources are available, the optimal cost-effectiveness path is from test-and-treat to the combination strategy of test-and-treat and PrEP (25% of high-risk MSM); followed by the same combination strategy of test-and-treat and PrEP, but with higher PrEP coverage.
		Scenario 2: PrEP for high-risk MSM with coverage of 25%, 60% PrEP effectiveness and 37% testing rate.	S2: $17,277.				
		Scenario 3: PrEP for high-risk MSM with coverage of 50%, 60% PrEP effectiveness and 37% testing rate.	S3: $17,979.				
		Scenario 4: PrEP for high-risk MSM with coverage of 75%, 60% PrEP effectiveness and 37% testing rate.	S4: $18,452.				
		Scenario 5: PrEP for high-risk MSM with coverage of 25%, 60% PrEP effectiveness and 90% testing rate.	S5: $13,835.				
		Scenario 6: PrEP for high-risk MSM with coverage of 50%, 60% PrEP effectiveness and 90% testing rate.	S6: $16,636.				
		Scenario 7: PrEP for high-risk MSM with coverage of 75%, 60% PrEP effectiveness and 90% testing rate.	S7: $18,110.				
		Scenario 8: PrEP for high-risk MSM with coverage of 25%, 60% PrEP effectiveness, 90% ART utilization rate for all diagnosed PLWH and 90% testing rate.	S8: $7,574.				
		Scenario 9: PrEP for high-risk MSM with coverage of 50%, 60% PrEP effectiveness, 90% ART utilization rate for all diagnosed PLWH and 90% testing rate.	S9: $10,485.				
		Scenario 10: PrEP for high-risk MSM with coverage of 75%, 60% PrEP effectiveness, 90% ART utilization rate for all diagnosed PLWH and 90% testing rate.	S10: $12,218.				
Hu et al. (32)	Scenario 1: non-ART.	Scenario 2: standard-ART.	S2: $28,272.	Not included	Not included	Not included	Early-ART and early-ART plus partners' PrEP were cost-effective (parameters included in the sensitivity analysis had a minimal impact)
		Scenario 3: early-ART.	S3: $12864.				
		Scenario 4: non-ART plus partners' PrEP (in which participants received medical care without ART and all of their sexual partners were assumed to take daily PrEP).	S4: $47321.				
		Scenario 5: standard-ART plus partners' PrEP (in which participants received ART 13–36 months post-infection, and all sexual partners were assumed to take daily PrEP until their partners reached un- detectable VL).	S5: $38,287.				
		Scenario 6: early-ART plus partners' PrEP (in which participants received ART within 12 months post- infection, and all sexual partners were assumed to take daily PrEP until their partners reached undetectable VL).	S6: $16,817.				

*S, scenario; ART, antiretroviral therapy; PrEP, pre-exposure prophylaxis; HIV, human immunodeficiency virus; HRMSM, high-risk men who have sex with men; MSM, men who have sex with men; M-C-P, more-casual-partner; M-S-P, more-steady-partners; QALY, quality-adjusted life years; ICER, incremental cost-effectiveness ratio; LY, life year; DALY, disability-adjusted life years; VL, viral load; PLWH, people living with human immunodeficiency virus; person-y, person-year*.

### Quality Assessment

To critically assess the methodological rigor of the economic evaluation of each article, the 24-item checklist Consolidated Health Economic Evaluation Reporting Standards (CHEERS) developed by Husereau et al. (29) was used. This checklist was chosen due to its robustness and authoritativeness. Two investigators (YQM and YHZ) independently assessed each item on the checklist for all studies, and any disagreements were discussed until a resolution was reached. The full quality assessment can be found in the supporting information: Supplementary Table 1.

## Results

**Figure 1:**
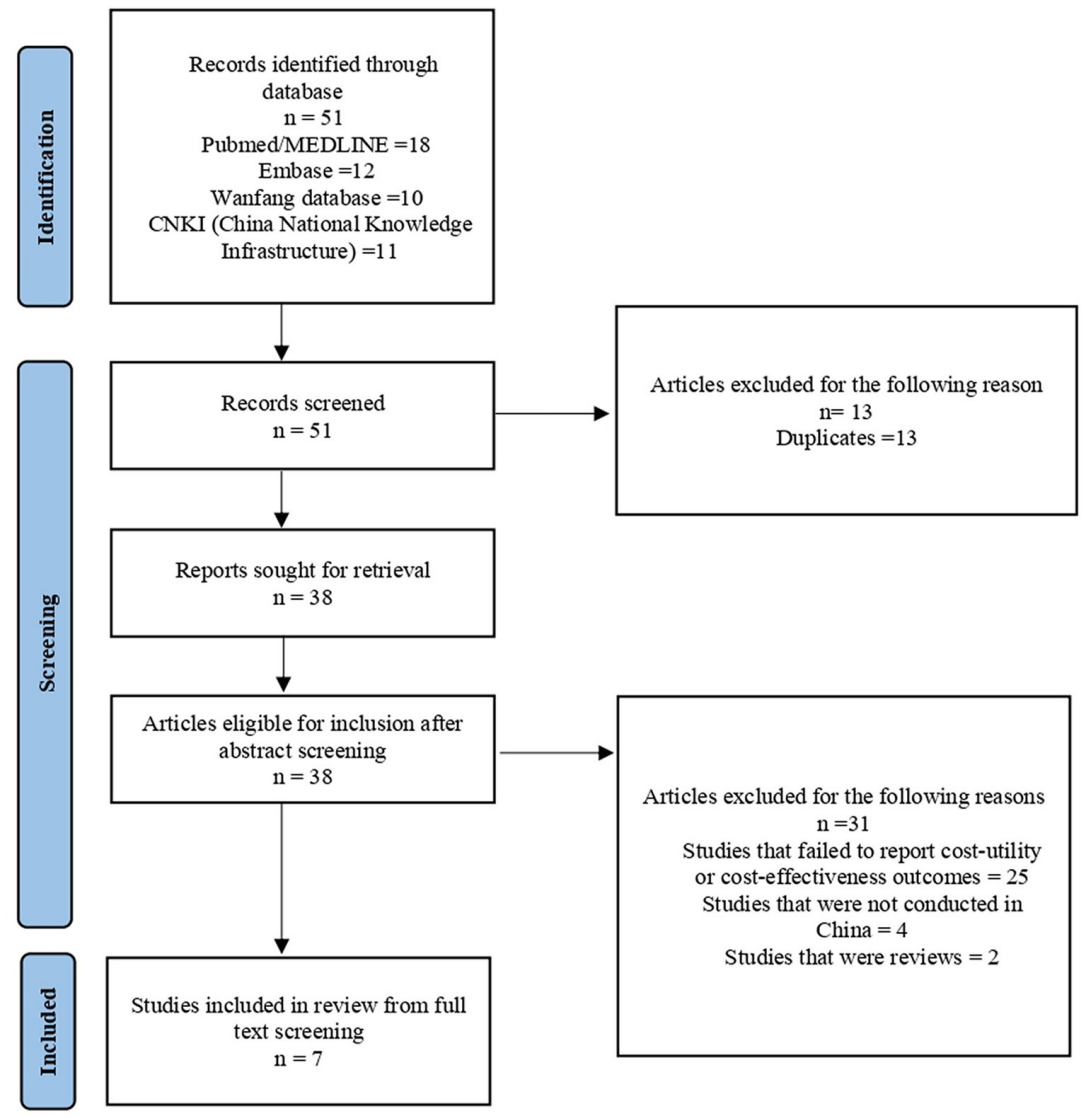
Flow diagram of selection process.

A total of 38 unique references were initially identified by our searches. After screening, seven study reports on CE were retained and included in the review. A summary of the study search and selection process through the review is presented in Figure 1.

The characteristics of the included studies are summarized in Table 2. Two out of seven studies (29%) were conducted using the Markov model (28, 30), four out of seven studies (58%) applied the compartmental model (31–34), and one study applied the risk-equation model (35). Four out of seven studies (58%) gave a specific definition of high-risk MSM (HRMSM) (31–34), based on four aspects: (1) annual number of sexual encounters (14.4–51.2/year) (33), (2)whether the anal sex occurred with a condom, (3) diagnosis of an STI (31), and (4) annual number of sexual partners (8–20/year) (31–34). Among all studies, only one study modeled PrEP implementation specifically among HRMSM (31). The model timeframe in three out of seven studies (43%) (32, 33, 35) was ≤ 10 years, and the other four of the seven studies (58%) (28, 30, 31, 34) used a timeframe longer than 10 years. Among all the drug regimen models, two out of seven studies (29%) (28, 30) used only TDF as the PrEP drug, three out of seven studies (43%) (33–35) used TDF/FTC (Truvada), one study (14%) (31) used TDF, TDF/FTC (Truvada) and TDF/3TC, and one study (14%) (32) did not specify the drug regimen. In addition to including the PrEP intervention in the models, four studies (58%) (30, 32, 34, 35) combined PrEP with other interventions: (1) standard HIV intervention strategies (program scale not specified), including HIV testing, risk-reducing counseling, condom distribution, STI management (30); (2) test and treat strategy (32, 34, 35); and (3) expanded volunteer counseling and tests (34).

The cost and impact assumptions of the included studies are summarized in Table 3. The majority of studies presented costs for PrEP implementation, including both drug costs and service costs (monitoring costs), except for two of the seven studies (29%) (28, 30) that included drug costs only. Assumptions of the annual cost of the drug ranged from the current price of tenofovir disoproxil fumarate (TDF) (between $20.5 to $1,804.5), Truvada (TDF/FTC) (between $1,843.8 to $8,327.8), and tenofovir/lamivudine (TDF/3TC) ($1,039.5) to zero cost. Except for one study (14%) (28) that set the drug costs lower than service costs, other cost estimates were driven by the cost of the drugs. All studies included averted ART costs. The estimated cost of ART determined by the time of use post-infection was between $473 and $16,761 per person/year. PrEP effectiveness estimates had wide ranges (from 23 to 100%), and one study (14%) (32) assumed that different PrEP effectiveness implied different adherence. Behavioral changes included reducing condom use (33, 34), increasing the number of sexual partners (32, 33), and reducing PrEP usage (32). One study (14%) (32) set a condition of behavioral changes in the primary analysis, and two studies (29%) (33, 34) included behavioral changes in the sensitivity analysis. Drug resistance and toxicity were not considered in all studies. Six out of seven studies (86%) conducted sensitivity analysis. Six studies (86%) applied a discount rate, among which four studies (58%) (28, 31, 34, 35) applied a discount of 3%, one study (14%) (32) applied 3.5% and another study (14%) (33) applied 5%.

Descriptions of all modeled scenarios and CE estimates are summarized in Table 4. All seven studies conducted (100%) a cost-utility analysis with a cost/QALY gained or cost/DALY averted as the outcome measures, as well as performing a CE analysis including survival as the effective measure (28, 30–35).

Among MSM without prioritization, one study (14%) (28) found that TDF was cost-effective with a price far lower than the current market price, while two studies (29%) (30, 33) found that PrEP was cost-effective only if TDF was reduced by 5.5% and TDF/FTC was reduced by 40% from the current price. When targeting HRMSM or serodiscordant couples, two studies (29%) (34, 35) found that PrEP was cost-effective when combined with interventions such as the test-and-treat strategy. Among HRMSM, one study (14%) (31) found that TDF or TDF/3TC was cost-effective, another study (14%) (33) found that TDF/FTC was cost-effective, and a third study (14%) (33) found that TDF/FTC would be cost-effective with a market price reduction of 50%. However, only three studies (43%) found that implementing other interventions, such as test-and-treat strategies and standard HIV intervention strategies without PrEP, was cost-effective (30, 32, 34). Two studies (29%) (30, 32) found that PrEP would not be cost-effective in all scenarios with the current market price.

## Discussion

To our knowledge, this is the first systematic review to summarize economic evaluations of PrEP implementation among MSM in China. A total of seven modeling studies on CE analysis of PrEP were identified in Chinese MSM.

### Identification of the Target Population Among MSM

Studies have simulated the CE of PrEP in different target populations among MSM. One of the simulation methods was to apply PrEP indiscriminately among all MSM. For example, Fan et al. (30) applied PrEP to all MSM populations without any restrictions, Zhong et al. (28) set an age minimum of MSM populations older than 14 years old, and Hu et al. simulated PrEP among HIV-negative MSM in serodiscordant couples. Another simulation method selected HRMSM and applied a population mixing pattern. Among them, Wong et al. (32) defined MSM with more than eight sex partners per year as HRMSM and assumed that they account for 43% of the MSM population, Li et al. (34) defined the HRMSM as those who have an average of 15 sex partners per year, which accounted for 20% of the MSM population, while low-risk men who had sex with men were defined as having 2.6 sex partners per year. Meanwhile, Zhang et al. (31) limited the study population to HRMSM and assumed that they accounted for 30% of the population of MSM in China. Except for those who had more than ten sexual partners every six months, MSM who reported condomless anal sex or were diagnosed with an STI in the past 6 months were also defined as HRMSM in Zhang's study (31). Wei et al. (33) further divided HRMSM into MSM with more casual partners (M-C-P) and MSM with more steady partners (M-S-P). The study compared the CE of PrEP modeling among each population, with each type of population having a different number of sex partners.

Prioritization determined by sexual activity characteristics to deliver PrEP to MSM at higher risk of HIV exposure was proven to be cost-effective by the included studies. The highest-risk population, HIV-negative MSM in a serodiscordant couple, was shown to achieve the most CE in multiple studies around the world (9, 10), which was consistent with the findings of Hu et al. (35). However, targeting all HRMSM is not necessarily a cost-effective approach. We concluded that targeting HRMSM with M-C-P as defined by Wei et al. (33) was cost-effective, which was consistent with the findings of Schneider et al. (9) in Australia. At market prices in 2016, a coverage of 25% [HRMSM defined by Li et al. (34)] to 30% [HRMSM defined by Wong et al. (32)] in the HRMSM was also proven to be cost-effective. In the US, studies targeting HRMSM at a relatively young age, which provided high coverage in HRMSM, indicated a high incremental cost-effectiveness ratio (ICER) [$US 31,970 per QALY gained in Desai et al. (36) and $US 298,000 per QALY gained in Paltiel et al. (37)]. This suggested that PrEP should be implemented among a specific population of MSM at low coverage. However, current studies in China omit the identification cost of this specific group, which results in mixed results. Moreover, the consensus for medication guidance in China suggested that HIV self-test results cannot be a sufficient basis for PrEP initiation because of the inability to rule out the possibility of an acute period of infection (17). Therefore, clinical assessments of HIV infection status are needed. Future modeling studies should include the identification cost more precisely.

Since the definition of which subpopulation of MSM should be PrEP targeted in the official document used in China refers to international guidelines, Hall et al. (11) conducted a cohort study evaluating the effectiveness of current international guidelines in identifying the PrEP targeted population in China. These findings implied that international guidelines are hardly useful in defining the targeted population in China and called for PrEP as a prevention strategy for anyone at “substantial risk,” which is similar to the PrEP recommendation from the WHO (38). Consequently, future studies should include more clinical data to propose better clinical decisions for decision-makers, identify and meaningfully engage those at highest risk according to the national guidelines that suit China's current socio-medical status to maximize HIV prevention.

### Treatment Heterogeneity

The expert consensus on pre-exposure prophylaxis from Chinese authorities (17) and from the WHO (39) both suggested using oral TDF/FTC (Truvada) (300 mg/200 mg) as the best regimen for its mild side effects. Among the studies in our review, the average price of daily TDF/FTC was set to $5,600 per person-year [Wei et al. (33): $8,327, Zhang et al. (31): $3,457, Li et al. (34): $6,909, Hu et al. (32): $3,706], and event-driven TDF/FTC was set to $1,843.8 [Zhang et al. (31): $1,844]. Compared to the threshold for a cost-effective intervention for a DALY averted, QALY saved, or LY saved (e.g., the threshold in 2017 was $7,723), and all CE analyses concluded on a negative CE result regarding implementing daily TDF/FTC as the official drug in China to target MSM without prioritization (with the cost of more than $60,202 per QALY saved). Zhang et al. (31) compared multiple types of PrEP drugs and concluded that the cost of PrEP needs to be below a threshold of $1,700 (¥11,305) per person/year to be cost-effective among HRMSM at 50% coverage, which is a 50% reduction on the modeled price ($3,457, ¥22,990 per person-year) of daily Truvada. Wei et al. (33) concluded that 60% of the current price (ideal price: $4,996, ¥33,228 per person-year) of the modeled international market price was $8,328 (¥55,380) per person/year for MSM with no prioritization and 80% (ideal price: $6,677, ¥44,404 per person/year) for HRMSM with MSP at 20% coverage and the current price for HRMSM with MCP. With no specified drug, Wong et al. used the international market price of Truvada ($7,880, ¥52,402 per person-year) and concluded that a 93% price reduction (ideal price: $519, ¥3,451.4 per person-year) on PrEP at 30% coverage among MSM without prioritization was cost-effective. However, policies have changed dramatically and have caused consequent changes in drug prices. Truvada (the branded drug of TDF/FTC) has a very high international price (¥4615, $694/30 tablets) (33). In 2017, it was included in China's medical insurance (applied only to people living with HIV), and the national market price changed to ¥1,905, $286/30 tablets in 2017 (40) [the same as the price modeled in the study by Zhang et al. (31)]. In June 2020, the first domestic generic TDF/FTC by Jiangsu Chia Tai-Tianqing Pharmaceutical Co., Ltd. was developed, and its market price was ¥1,180/30 tablets (41) in 2020 and ¥980 and $153/30 tablets (42) in 2022. In 2021, another generic TDF/DTC was developed by Anhui Baker Biopharmaceutical Co., Ltd., and the price of TDF/FTC dropped to ¥286, $45/30 tablets. Such changes make TDF/FTC cost-effective for MSM at 20% coverage with no prioritization in 2017 according to Wei et al. (33) and at 30% coverage in 2021 by Wong et al. (32) and among HRMSM at 50% coverage in 2021 according to Zhang et al. (31). Except for studies that only targeted HRMSM, CE could be achieved using the current market price of generic TDF/FTC among all MSM according to the remaining modeling studies. Therefore, future studies should include all types of MSM to analyze the different PrEP program scales between MSM and HRMSM.

With the market price far lower than Truvada, daily TDF and daily TDF/3TC were thought to be alternatives (31). In February, the National Medical Products Administration authorized the domestic generic TDF, and its market price changed from ¥132, $20/30 tablets (branded drug) in 2013 to ¥83.4, $13/30 tablets in 2016 (43) [generic drug, same as the modeled price by Zhong et al. (28)]. Except for Zhong et al. (28), who based the inference that PrEP can save $2,718 per QALY gained with 94% adherence on the assumption that TDF (event-driven) purchased from CDC directly costs only $21 per person/year, other studies set the average market price of daily TDF to be the international market price: $1,295 [Fan et al. (30): $1,805, Zhang et al. (31): $786], Fan et al. (30) concluded that TDF targeting MSM without prioritization needs a 5.5% reduction (ideal market price: ¥11,340, $1705) on the market price in 2016 to achieve CE, which could be achieved due to the price change in 2016, while Zhang et al. (31) found that TDF is cost-effective with 50% coverage on HRMSM.

The US President's Emergency Plan for AIDS Relief (PEPFAR) Scientific Advisory Board recommended TDF/3TC as an acceptable alternative to TDF/FTC for PrEP in December 2015 (44), and the WHO recommended the interchangeability of TDF/FTC and TDF/3FC in 2016 (15). Most countries recommending the use of 3TC/TDF as PrEP are in Sub-Saharan Africa, where the use of this regimen saves $10 per person/year compared to TDF/FTC (44). Domestic generic TDF/3FC was authorized by the National Medical Products Administration in 2019, with no branded drug imported, the market price of which was ¥898/30 tablets (45). Only one study included generic TDF/3TC (daily) with a higher modeled price [Zhang et al. (31): $1,039 per person-year] and demonstrated its CE among HRMSM at 50% coverage. Therefore, future studies should investigate its CE among MSM without prioritization at higher coverage.

Apart from the drug types mentioned above, new regimens with clinical effectiveness demonstrated for PrEP have emerged. F/TAF with fewer side effects than TDF/FTC (46) (a combination of emtricitabine 200 mg and tenofovir alafenamide 25 mg) was approved for PrEP in February 2020 in the US, among men and transgender women, excluding persons who conduct vaginal sex (47) with an international market price of ¥27,360, $4145/30 tablets (45). Its generic drug was authorized in October 2021 in China but is currently not on the market. Additionally, an injected PrEP drug that only requires 1 dose/60 days called Apretude (cabotegravir, CAB-LA) was approved in the US (8).

In high-income countries such as the US, the PrEP program used branded TDF/FTC costs from $107,000 to $303,091 per QALY saved among MSM without prioritization due to the high cost of drugs used for PrEP (US$8,000 to US$9,300 per person-year for PrEP drugs only) (48). Generic TDF/FTC in the US was approved in 2020 with a market price lower than branded TDF/FTC (49). A recent modeling study in the US found that the improved clinical benefits of branded F/TAF are worth no more than the additional cost of $370 of PrEP per person/year compared to generic TDF/FTC among MSM without prioritization (50), and the negative results accord with the latest modeling study that targeted very high-risk MSM and transgender women (46). However, both studies have proven generic TDF/FTC to be cost-effective among MSM without prioritization. The latter study, which also included injected PrEP, concluded that oral PrEP limits the additional price that society should be willing to pay for CAB-LA (46). For countries such as Ireland, where several generic TDF/FTC have been licensed and marketed for use since 2018, the CE has also been demonstrated among HRMSM under the current market price of $912 (2018 US$) per person-year, with the ICER in all scenarios below $7,150/QALY (51).

In comparison, in low-income countries such as Peru, PrEP with a price of $420 to $600 per person/year could be a cost-effective addition to current prevention programs for MSM populations (up to US$1,702/DALY averted) (48). For middle-income countries such as Israel, PrEP was included in the official drug registry in 2017 with a market price of $6,887 per person-year. Studies have suggested that neither daily TDF/FTC ($967,744/DALY averted) nor on-demand ($475,673/DALY averted) are cost-effective among HRMSMs unless there is a 90.7% price reduction in the current market price. However, after negotiations in January 2020 between the Israeli Ministry of Health and pharmaceutical manufacturers to introduce PrEP into the National Basket of Health Services at a greatly discounted price, CE would be achieved, as estimated by the underlying model of this research (52).

There is a huge difference in the price setting of the same drug among different studies. Despite the fact that drug prices fluctuate and the references for price-setting from past literature can differ from year to year, policy changes and the emergence of domestic generic drugs from different manufacturers (41, 45, 53) can contribute to dramatic drug price changes as well. However, changes in drug prices and policy reforms across the world in recent years have focused on making PrEP increasingly cost-effective. In addition, most modeling studies in China did not consider the toxicity and drug resistance of the different drug types of PrEP. To establish the most cost-effective PrEP drug type, future studies should consider not only the monitoring cost but also the potential treatment cost caused by the side effects of different types of PrEP drugs. Moreover, interventions in the included studies tended to be bundled, thus obscuring the effect of each approach. Consequently, holistic study designs are required to disentangle the single effect of each implementation component of the combined interventions, such as the test-and-treat strategy with PrEP, from their combined effect.

### Research Indication and Future Direction

Our study indicated that the identification of HRMSM to be targeted and a reduction in the current market price of PrEP drugs are the two most limiting factors to achieve the CE of PrEP. However, all studies that included the cost of PrEP used prices from 2016 to 2017, when the market prices of PrEP were changing. After 2020, new generic drugs have emerged in China, and the market price of PrEP drugs has decreased dramatically, thus achieving CE of PrEP among MSM without prioritization as estimated by the underlying model of multiple studies (30–33), while all of these studies originally concluded negative results. Despite the absence of a specific program scale in two studies (28, 30), a general conclusion for decision-makers in China can still be reached (generic TDF/FTC: among MSM without prioritization at 20–30% coverage, among HRMSM at 50% coverage; TDF: among MSM without prioritization combined with standard HIV intervention; among HRMSM at 50% coverage; TDF/3FC; among MSM without prioritization combined with standard HIV intervention). Therefore, future studies should include the latest price changes while applying a larger scale of PrEP among MSM in CE analysis. Additionally, TDF or TDF/3CT with a relatively low market price could be an alternative for the recommended drug TDF/FTC when targeting HRMSM, but CE analysis, including the difference in its clinical effect from TDF/FTC, is needed. Furthermore, the invention of new drugs such as F/TAF and CAB-LA should also be considered in future CE analysis of PrEP in China.

However, analyzing the included studies revealed a lack of standard and transparent methods in the modeling studies. The heterogeneity of interventions used in each study does not facilitate the comparison of scenarios in different studies or the weighing of policy alternatives. Modeling studies should aim to simulate the current situation and decision context closest to real-world settings (54). However, models based on untested assumptions could lead to false expectations of implementations. Despite the existence of tools for evaluating the quality of health economic modeling studies (29), the lack of tools for evaluating model assumptions and their results calls for a standard method. Modelers should not only address the rationale for applying their model calibration, to strengthen the quality of the data applied but also interpret the results in a manner as simple as possible.

Moreover, there are significant gaps in awareness of PrEP, willingness to take PrEP, the actual uptake of PrEP, and adherence to PrEP among Chinese MSM (21). To facilitate this process, researchers have demonstrated the effectiveness of participatory approaches such as crowdsourcing PrEP promotion (55). For example, an HIV peer-educational program targeting 1,697 recipients (MSM and transgender women) in Thailand successfully improved community awareness and initiation of PrEP uptake. However, community-based health education or intervention programs of PrEP on this scale have rarely been conducted in China, nor have studies of health economic evaluations of such behavioral-altering programs been conducted. Future studies should emphasize the effect of health promotion programs regarding PrEP and their synergy with other biomedical interventions in modeling studies to provide more evidence to policymakers.

Furthermore, in the event of a public health emergency such as the COVID-19 pandemic, there may have been some obstacles for patients to continue PrEP treatment. Initiating follow-up every 3 months is recommended in China's PrEP guidelines (17), but when face-to-face follow-up cannot be easily achieved, Tele-PrEP is recommended (56). With Tele-PrEP, follow-up visits can be performed *via* video conferencing. Patients can self-administer home specimen collection kits and mail back dried blood spot filter paper specimens for virtual follow-up (17), but the cost of mailing and self-test kits could impose additional fees on patients. Additionally, monitoring of medication compliance can be performed electronically, such as a medication reminder electronic pillbox, online medication reminder notifications, and other services (17). The current market price of HIV and other STI self-test kits ranges from $14 to $43 (57). Based on the standards from the guidelines, the cost of a follow-up test could be at least $57 per person/year, which is higher than the service cost (¥95 per person/year) modeled in a study by Zhang et al. (31). Additionally, concerns regarding the quality of testing kits bought online remain a major obstacle that makes patients reluctant to test (57). Additionally, the market price of some domestic generic drugs could be unpredictably affected by the intermittent pandemic outbreaks in China. For example, the market price of domestic generic TDF/FTC produced by Anhui Baker Biopharmaceutical Co., Ltd. dropped from ¥286, $43/30 tablets to ¥249, $38/30 tablets in July (42), and the price of the same generic drug produced by Jiangsu Chia Tai-Tianqing Pharmaceutical Co., Ltd. in 2021 fluctuated from ¥1,080/30 tablets (July) to ¥666, $104/30 tablets (October), and then ¥980, $153/30 tablets (November) (41). Patients taking PrEP may not be familiar with the changing pace of market prices or policies in the absence of official platforms keeping track of such information; consequently, they may miss doses or even stop taking medication. Therefore, in this post-pandemic era, initiating market price reduction while improving the quality standards of self-test kits according to the guidelines and improving remote monitoring platforms for medication compliance have become priorities.

Last, some studies omitted the existence of marginal cost and assumed the relationship between program scale and its cost to be linear correlates, which results in certain inaccuracies in their CE evaluation. Guinness et al. (58) developed a function stating that the shape of costs of HIV prevention programs can be thought of as a “U”-shaped curve with increasing coverage, suggesting a minimum value that indicates the most cost-effective program scale. Wei et al. (33) found that the marginal return of PrEP implementation decreases with the increase in its coverage, which was consistent with the findings of Guinness et al. (58) and Juusola et al. (59). The shape of the cost function can reveal very different unit costs, and thus very different CE, at different scales of implementation. However, few studies are conducting the precise cost function of HIV prevention to determine the optimal program scale in China. Consequently, further investigation should be made regarding the detailed effects of more PrEP program scale levels to better inform the policymakers of the most cost-effective coverage and intervention.

### Limitation

There are several limitations to this review. First, the review included a heterogeneous set of studies with different model assumptions. Consequently, straightforward comparisons and a conclusion on the best drug type based on current studies were infeasible. Therefore, this review focused on study description and comparison instead of quantitative analyses. In addition, as some studies contain no information about the particular dosage of each PrEP regimen, our study did not specify such information. Since some of the studies evaluated CE in terms of Chinese yuan, we converted values into US dollars using the exchange rate of the year in which the study was published for a more accurate comparison; however, exchange rates constantly fluctuate within a year, so we omit this minor but existent detail.

Another limitation is that during the review process, because of the scarcity of modeling studies on PrEP among Chinese MSM, studies were directly compared as long as they contained CE analysis. For example, Zhong et al. (28) set the drug to be purchased from the CDC significantly reduced the price of the drug at the time of modeling and therefore was not directly comparable to other models that used current market price as a parameter. The small-scale modeling studies combined with real-world studies conducted by Hu et al. (35) would normally be excluded from systematic reviews of CE analysis because of their unrepresentativeness. Thus, this research does not yield very specific policy recommendations for China (such as the specific amount of price reduction for PrEP drugs and the specific scale of implementation) but only some policy directions for improvement.

## Conclusion

We found that PrEP is only cost-effective when using TDF or TDF/3TC regimens. Under the current market price, TDF/FTC is only cost-effective when targeting HRMSM with M-C-P or 50% HRMSM with M-S-P. A reduction of at least 5.5% in the PrEP current market price or a combination of PrEP with other HIV preventive approaches would be cost-effective for MSM without prioritization. However, after including price changes in recent years, TDF/FTC is thought to be cost-effective among MSM without prioritization at 20–30% coverage and among HRMSM at 50% coverage.

Nevertheless, a number of observations can be made about the state of the literature. First, PrEP is projected to be more cost-effective when paired with efforts to identify infected individuals through expanded testing or the test-and-treat strategy. However, the lack of studies on ongoing community-based health interventions results in a lack of information on the wider prevention impact of PrEP as part of a package of combination prevention modalities. In addition, current studies seldom use consistent standards when conducting different scenarios in model design and assumption. Moreover, future studies need to clarify the relationship between cost and coverage (scale) among different programs to provide a well-defined vision for policy-makers. Last, more studies need to be performed to identify the most cost-effective subpopulations for targeted coverage given China's current socioeconomic status and the latest market price changes of PrEP.

## Data Availability Statement

The original contributions presented in the study are included in the article/Supplementary Material, further inquiries can be directed to the corresponding author/s.

## Author Contributions

YM, YZ, and MZ conducted the search, selection of records, and data extraction. Quality appraisal was conducted by YM, MZ, PW, and FC. All authors drafted the systematic review protocol. All authors have read and approved the final manuscript.

## Funding

This work was supported by grants from the National Natural Science Foundation of China (Project No. 71874100), Beijing Municipal Science & Technology Commission (Project No. D171100006717002), and AbbVie Pharmaceutical Trading (Shanghai) Co., Ltd. The funder was not involved in the study design, collection, analysis, interpretation of data, the writing of this article or the decision to submit it for publication.

## Conflict of Interest

The authors declare that the research was conducted in the absence of any commercial or financial relationships that could be construed as a potential conflict of interest.

## Publisher's Note

All claims expressed in this article are solely those of the authors and do not necessarily represent those of their affiliated organizations, or those of the publisher, the editors and the reviewers. Any product that may be evaluated in this article, or claim that may be made by its manufacturer, is not guaranteed or endorsed by the publisher.
